# The human gut virome: composition, colonization, interactions, and impacts on human health

**DOI:** 10.3389/fmicb.2023.963173

**Published:** 2023-05-24

**Authors:** Evan Pargin, Michael J. Roach, Amber Skye, Bhavya Papudeshi, Laura K. Inglis, Vijini Mallawaarachchi, Susanna R. Grigson, Clarice Harker, Robert A. Edwards, Sarah K. Giles

**Affiliations:** Flinders Accelerator for Microbiome Exploration, College of Science and Engineering, Flinders University, Bedford Park, SA, Australia

**Keywords:** bacteriophage, fecal transplant, virus, gut-brain axis, viral dark matter

## Abstract

The gut virome is an incredibly complex part of the gut ecosystem. Gut viruses play a role in many disease states, but it is unknown to what extent the gut virome impacts everyday human health. New experimental and bioinformatic approaches are required to address this knowledge gap. Gut virome colonization begins at birth and is considered unique and stable in adulthood. The stable virome is highly specific to each individual and is modulated by varying factors such as age, diet, disease state, and use of antibiotics. The gut virome primarily comprises bacteriophages, predominantly order Crassvirales, also referred to as crAss-like phages, in industrialized populations and other *Caudoviricetes* (formerly *Caudovirales*). The stability of the virome’s regular constituents is disrupted by disease. Transferring the fecal microbiome, including its viruses, from a healthy individual can restore the functionality of the gut. It can alleviate symptoms of chronic illnesses such as colitis caused by *Clostridiodes difficile*. Investigation of the virome is a relatively novel field, with new genetic sequences being published at an increasing rate. A large percentage of unknown sequences, termed ‘viral dark matter’, is one of the significant challenges facing virologists and bioinformaticians. To address this challenge, strategies include mining publicly available viral datasets, untargeted metagenomic approaches, and utilizing cutting-edge bioinformatic tools to quantify and classify viral species. Here, we review the literature surrounding the gut virome, its establishment, its impact on human health, the methods used to investigate it, and the viral dark matter veiling our understanding of the gut virome.

## 1. Introduction

The idea ‘all disease begins in the gut’ is attributed to the ancient Greek physician Hippocrates nearly 2,000 years ago. While we did not have the scientific tools to explore this hypothesis more then, it is now clear that the gut has a role in overall physiological health. The most prevalent type of virus within the gut virome are bacteriophages (Phage; Edwards et al., 2019; Shkoporov et al., 2019), viruses that infect bacterial species. Phages were first identified in 1915 and 1917 by Frederick Twort, and Felix D’Herelle, respectively, and we are only now beginning to uncover the roles of phages in human health (Taylor, 2014; Barr, 2017). Advanced sequencing techniques and computational analysis have played a role in discovering novel phages and their role within the gut virome (Columpsi et al., 2016; Yutin et al., 2021).

The gut virome comprises many species of interest to the microbial and medical science communities. Viruses have played an integral role in human biology for as long as we have existed. The gut virome is increasingly important in understanding human health and fundamental biological ecology (Van Espen et al., 2021). The colonization of the gut virome is proposed to start at birth and is modulated by a range of factors to create a profile unique to each individual (Liang and Bushman, 2021). The common constituents of a stable virome can be disrupted by disease; for example, Crohn’s, ulcerative colitis, and diabetes can all contribute to gut dysbiosis. Gut dysbiosis is an imbalance in the microorganisms within the gut microbiome (bacterial component), which can negatively impact human health. For example, *Caudoviricetes,* a class of tailed phages, are noticeably altered in diversity and richness in dysbiotic people compared to healthy controls (Fernandes et al., 2019; Yang et al., 2021). Transferring the fecal microbiome, including its viruses, from a healthy individual can restore the functionality of the gut and alleviate the symptoms of some chronic illnesses (Aira et al., 2021).

Here we consider our current understanding of the enteric virome, including its establishment in early life, its impact on our overall health, and the possible influences on mood disorders. Existing tools being used to illuminate viral dark matter, viral species that are undefined or not well understood, and directions for further research are also discussed.

## 2. The enteric virome in early life

### 2.1. The virome established at birth

Birth is a major life event and where the seeding of the human virome begins. For babies born by either cesarean section (CS) or vaginal delivery (VD), it is generally accepted that viral colonization begins immediately upon exposure to a non-sterile environment *post-utero* and that the mode of birth affects the diversity of the viruses and phage communities present (McCann et al., 2018; Beller and Matthijnssens, 2019). However, early microbial colonization is a topic of some debate. There were some suggestions the human microbiome (including the virome) may begin to be seeded pre-birth *in utero*, as bacterial, fungal, archaeal, and viral DNA have all been reported in meconium and amniotic fluids (Stinson et al., 2019). Unfortunately, these studies relied on the amplification of low biomass samples for 16S sequencing, and the findings were recently demonstrated to almost certainly be the result of contamination (Kennedy et al., 2023). These false positives probably arise from the contamination of biological reagents or samples, and where stringent controls were utilized, there were no significant differences between samples and negative controls (Lauder et al., 2016; Perez-Muñoz et al., 2017; Leiby et al., 2018; Lim et al., 2019). Any future studies should utilize these lessons learned and employ positive and negative controls, amplification-free sequencing where possible, and increased sample sizes.

**Figure 1:**
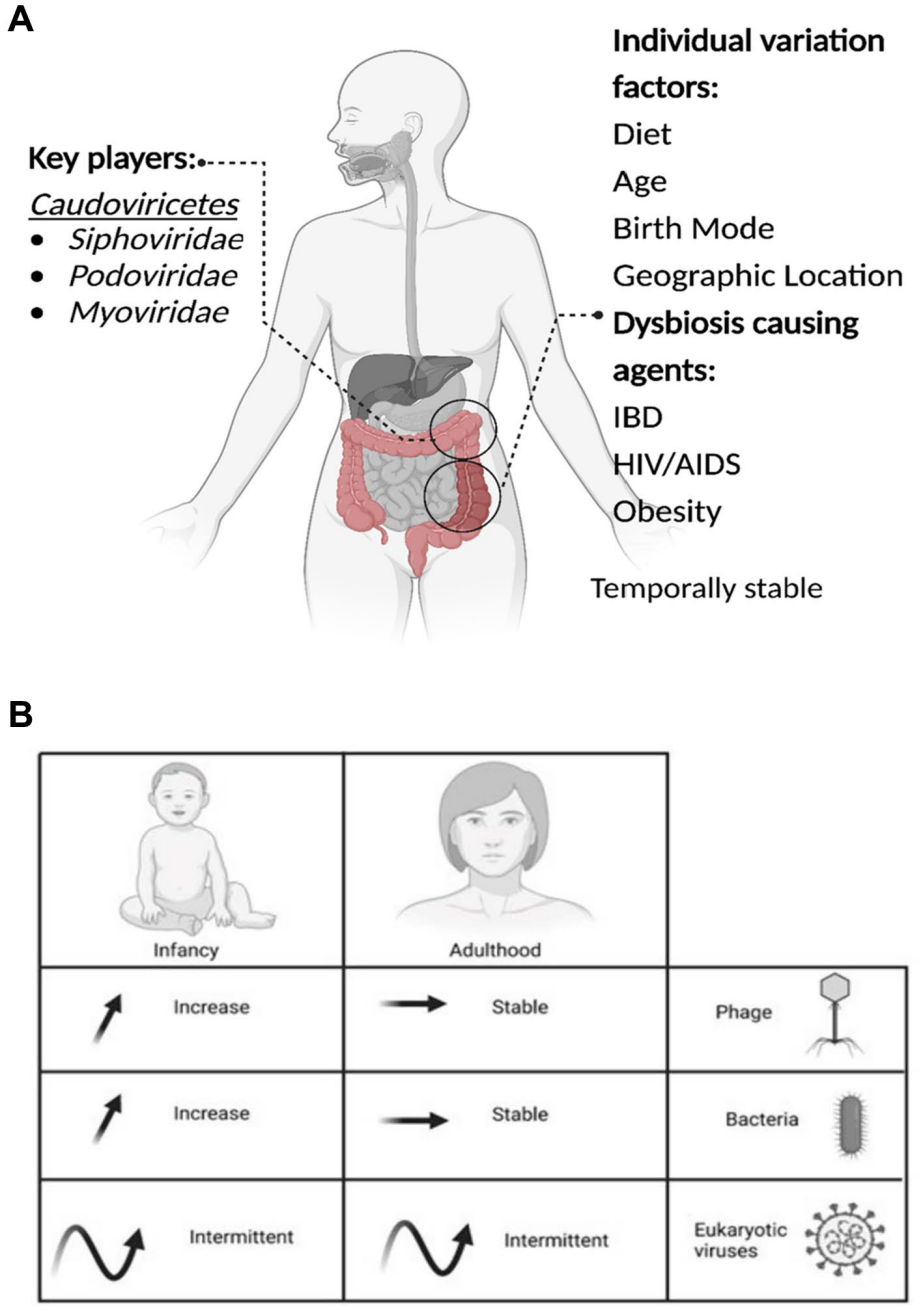
The important aspects of the gut virome. **(A)** Key players, Dysbiosis-causing agents, and Individual variation factors. **(B)** The microbial dynamics throughout life are presented in the table, the arrows represent the increase, stable or intermittent diversity of phages, bacteria and eukaryotic viruses, at different stages of life.

Method of birth creates an observable difference in the composition of viromes. Babies born by VD have a more diverse virome than those born by CS, with *Caudoviricetes*, *Microviridae,* and *Anelloviridae* the most abundant viruses detected (McCann et al., 2018). The effect of the mode of birth on microbial composition is another area of contention. Several studies utilizing bacterial 16S sequencing could not identify significant microbiome differences between birth modes (Barr, 2017; McCann et al., 2018; Beller and Matthijnssens, 2019; Maqsood et al., 2019). However, a recent longitudinal study of 1,679 gut microbiota samples from 310 CS and 281 VD babies demonstrates clear and significant differences in the microbiomes by mode of birth and differences resulting from prophylactic antibiotics pre-delivery (Shao et al., 2019). Following delivery, colonization of the infant’s gut microbiome is a dynamic process with the virome changing rapidly over at least the first two years, starting with a high phage richness that decreases over the first 24 months (Figure 1; Lim et al., 2019; Gregory et al., 2020; Dahlman et al., 2021; Taboada et al., 2021).

Nevertheless, it has been reported that viruses significantly increase in the infant’s virome after entering daycare (Beller et al., 2022). Post-initial colonization, the gut virome remains largely stable, with fluctuation increasing and decreasing over long periods (1–2.5 years; Minot et al., 2013). The next notable shift occurs after the age of 65, where changes in the virome likely follow shifts in the broader microbiome as its stability/fluctuation periods change (Nagpal et al., 2018; Gregory et al., 2020).

### 2.2. Breastfeeding shapes the gut virome

The mode of early feeding plays a significant role in the establishment of the human gut virome. Both eukaryotic viruses and phages are transferred from mother to infant via breastfeeding (Townsend et al., 2012; Pannaraj et al., 2018; Mohandas and Pannaraj, 2020), as a result, the composition of the early human gut virome varies according to whether a child is breastfed or formula-fed (Fragkou et al., 2021; Liang and Bushman, 2021). This difference in viromes is partly due to the maternal antibodies transferred from mother to infant via breastmilk, conveying additional immunity that is not transmitted through formula milk (Albrecht and Arck, 2020). In addition, breast milk also promotes the growth of specific bacteria, e.g. *Bifidobacteria, Lactobacillus,* and *Streptococcus* (Lawson et al., 2020; Lyons et al., 2020), as it contains a variety of complex macronutrients composed of lipids, proteins and carbohydrates, immune cells and antibodies, as well as human milk oligosaccharides. These lipids represent 40–60% of the energy in mature milk providing essential nutrients like polyunsaturated fatty acids, complex lipids (triacylglycerides, diacylglycerides, monoacylglycerides, free fatty acids, phospholipids, and cholesterol) (Boudry et al., 2021). The oligosaccharides make up the third most significant component of breast milk and are indigestible by the infant, such that they act as prebiotics for the gut microbiota (Andreas et al., 2015). However, interestingly, a mixed approach of both breast and bottle feeding creates a similar virome to a solely breastfed infant.

While breast milk is nutritionally and immunologically beneficial, it may also play a role in the transmission of non-beneficial viruses such as human immunodeficiency virus (HIV), hepatitis B virus, cytomegalovirus, Zika virus, and others (Blohm et al., 2018; Maqsood et al., 2021; Pang et al., 2021; Lawrence, 2022). Pathogenic bacteria can also be transferred from mother to infant via breast milk; cases of group B *Streptococcal* infection have been reported for many years (Kenny, 1977; Mantziari and Rautava, 2021). A further study into how the virome develops between the mother and infant may illuminate digestive, viral, and general health in early life. A study by Liang et al. (2020) on 676 samples has provided significant insight into how breast milk modulates the gut microbiome and virome in early life: a lack of breastmilk resulted in a lack of associated bacteria and subsequently an absence of phages. A study into the stepwise assembly of the gut virome should be conducted from birth up to 5 years of age, to provide an in-depth investigation into initial assembly and the ongoing stability of the gut virome.

## 3. The temporally stable virome

### 3.1. Virome diversity correlates with microbiome diversity

Enteric virome diversity is unique for each individual (as shown by mono- and di-zygotic twin studies; Moreno-Gallego et al., 2019) and is correlated with overall gut microbiome diversity. Most of the virome consists of phages (Minot et al., 2013; Shkoporov et al., 2019), and their replication dynamics are closely tied to bacterial populations and their replication (Breitbart et al., 2008).

Global assessment of the human virome revealed the existence of distinct viral enterotypes (Ogilvie et al., 2013; Camarillo-Guerrero et al., 2021), grouping into three broad categories defined by their prominent bacterial host (*Bacteroides, Prevotella,* and *Ruminococcus*) (Arumugam et al., 2014). However, this hypothesis should be examined using bioinformatics to reveal correlations between viral enterotypes and implications for human health.

### 3.2. Phage and prophage propagation and dynamics

Phages are intimately involved in the homeostasis of the gut microbiome. The balance between the phage’s lytic and lysogenic life cycles can impact this homeostasis and significantly affect human health (Mirzaei and Maurice, 2017; Garmaeva et al., 2019). In the lytic cycle, the phage infects a bacterium, hijacks its replication machinery, and causes the host cell to produce multiple copies of the phage. This replication leads to the bursting (lysis) of the host cell and the release of new phage particles, ready to infect other bacteria. This cycle is characterized by rapid replication, cell lysis, and host cell death. In the lysogenic cycle, the phage infects a bacterium and integrates its genetic material into the host’s genome, forming a prophage. While the prophage remains dormant, it does not cause lysis of the host cell; instead, it is replicated along with the host genome during cell division. The phage remains integrated into the host genome until an environmental signal or stressor triggers the switch to a lytic cycle (Mirzaei and Maurice, 2017; Garmaeva et al., 2019). Understanding the signals driving the balance between lytic and lysogenic life cycles is of paramount importance.

Many phages can reproduce via a lysogenic replication strategy; for example, in the gut, many observed bacteria carry prophage genes (Waller et al., 2014; Kim and Bae, 2018). These lysogenic prophages are proposed to follow the piggyback-the-winner (PtW) dynamic, which is often seen in high virus-microbe-ratio (VMR) environments (Knowles et al., 2016; Silveira and Rohwer, 2016; Paterson et al., 2019). Lytic phages, however, follow the traditional kill-the-winner (KtW) viral dynamic, seen commonly in lower VMR environments such as marine environments (Rodriguez-Brito et al., 2010). However, both PtW and KtW are seen in the human gut simultaneously (Silveira and Rohwer, 2016), likely due to the variety of phage species that exist and the hosts they infect being present at varying levels.

Lysogenic phages switch between lytic and lysogenic replication. The various signals that prompt switching (Howard-Varona et al., 2017), including biological factors such as cell development (Golding, 2011), phage latent periods (Abedon et al., 2001), and on a ‘majority rules’ basis upon infection by multiple phages in the same bacterial cell (Zeng et al., 2010). Multiple external signals, including antibiotics, Fe^2+^ levels, and DNA damage, also influence lysogeny/lytic switching in phages (Herskowitz and Hagen, 1980). The switch from temperate to lytic replication is usually observed as the bacterial cell lyses and releases phages into the environment. Interestingly, some phages can produce progeny virions while maintaining an integrated phage genome and cellular integrity. There are two known mechanisms for this, budding and extrusion; budding is where the phage pushes through the host cell wall and takes a portion of the membrane with them, and forms an envelope, *Plasmaviridae* can utilize this process. Extrusion, utilized by *Inoviridae*, is where the phage is squeezed out of the host through the cell membrane without acquiring an envelope (Dennehy and Abedon, 2021).

### 3.3. The gut virome changes throughout life

The enteric virome experiences large changes during the first 24 months after birth (Minot et al., 2013; Lim et al., 2015; Beller and Matthijnssens, 2019; Shkoporov et al., 2019). However, the enteric virome eventually settles into a unique composition for each individual and remains relatively stable with minor fluctuations. Reports have indicated that up to 80% of the sequences remain unchanged (Moreno-Gallego et al., 2019; Shkoporov et al., 2019). The gut virome’s temporal stability is integral to continual health. Gut microbiome stability has also been implicated in improved psychological well-being (Cryan et al., 2019; Mukhopadhya et al., 2019; Fulci et al., 2021).

The virome is involved in the function of many homeostatic processes and maintaining microbial populations through phage-bacterium interactions (Broecker et al., 2017; Halfvarson et al., 2017; Liu et al., 2017; Gogokhia et al., 2019; Ezra-Nevo et al., 2020). This stability is disrupted when the gut’s regular function is abnormal, and it enters dysbiosis (Broecker et al., 2016b). Increased diversity and richness of the virome may contribute to gut pathogenesis, as seen in inflammatory bowel disease (IBD; Norman et al., 2015; Zuo et al., 2019). The virome is also noticeably altered in the presence of other disease states, such as infection with *C. difficile*, type-1 diabetes, cancer, or AIDS (Broecker et al., 2016a; Park and Zhao, 2018).

### 3.4. Core species of the gut virome

The gut virome is comprised of eukaryotic and bacterial viruses of which phages make up the vast majority (Reyes et al., 2012; Minot et al., 2013; Edwards et al., 2019; Shkoporov and Hill, 2019). The human gut is thought to contain a core virome comprised of commensal viruses commonly found in the population. This concept was first raised by Willner et al. (2009) and more recently by Broecker et al. (2017), who show the virome to be relatively more stable than the bacterial component of the microbiome. Viruses of the class *Caudoviricetes* make up the majority of discovered gut phages (Garmaeva et al., 2019; Lawrence et al., 2019). When working bioinformatically with the phage genomes the collective term is the phageome (Manrique et al., 2016; Carding et al., 2017; Shkoporov et al., 2019), which is thought to be integral to human health through controlling bacterial mortality and microbial ecology (Krishnamurthy et al., 2016; Dahlman et al., 2021).

The most abundant phages in the human gut (at least, from industrialized populations) are a collection of phages of the order Crassvirales, also called crAss-like phages (Dutilh et al., 2014; Broecker et al., 2017; Shkoporov et al., 2018b; Honap et al., 2020; Koonin and Yutin, 2020). CrAss-like phages are known to infect bacterial species belonging to the abundant *Bacteroides* genus *B. intestinalis*, and *Bacteroides xylanisolvens* (Lim et al., 2018; Shkoporov et al., 2018a; Guerin et al., 2021). Another clade known as Gubaphage which is also prevalent across the globe, has a host range of *Bacteroides caccae*, *B. xylanisolvens*, and *Bacteroides vulgatus* among others (Camarillo-Guerrero et al., 2021). There is still a significant portion of the gut virome that remains uncharacterized. Identifying hidden species among this viral dark matter is a priority in viral metagenomics, and individual samples, as they could be playing a vital role in maintaining host health.

### 3.5. Rare and novel viruses

In addition to the core virome, rare viruses are also often reported in the human gut. It was originally reported that megavirus such as *Mimivirus*, *Marseillevirus*–viruses that infect amoebae–may be present within the human gut virome (Zuo et al., 2019). However, this erroneous result occurred due to insufficient filtering of sequence alignments (Sutton et al., 2020) and highlights the pervasiveness of false positives in virome profiling, which is discussed further on. Lak phages (Colson et al., 2013; Scarpellini et al., 2015; Hugon et al., 2017; Pires de Souza et al., 2021) known to infect *Prevotella* species (Devoto et al., 2019) are frequently reported. These viruses are also a constituent of the virome of many species beyond humans (Devoto et al., 2019; Tokarz-Deptuła et al., 2019). More research with special efforts to target uncommon viral species from the human gut should be undertaken to understand these rare virus species better.

### 3.6. Diet impacts the gut virome

Diet is a strong determinant of the human gut virome (Swidsinski et al., 2017; Schulfer et al., 2020). While inter-individual variation is the greatest differentiator of the human virome, those on similar diets develop convergent viromes (Minot et al., 2011). Changing diet can create a subtle but measurable shift in the gut virome of an individual and has been observed when fats, gluten, and alcohol intake has been altered (Schulfer et al., 2020; Zuo et al., 2020; Garmaeva et al., 2021). Dietary-induced shifts in the gut microbiome and virome of an individual may have an impact on human health through bacterial and phage compositional changes (Ezra-Nevo et al., 2020; Li et al., 2021), the precise mechanisms for how the virome changes and how it is influenced by dietary changes are not yet fully understood (Garcia-Gutierrez et al., 2020).

## 4. Gut dysbiosis

### 4.1. Inflammatory bowel disease

Inflammatory bowel disease (IBD) is a chronic disease with two dominant subtypes: ulcerative colitis, and Crohn’s disease (Zuo et al., 2019). Crohn’s disease can affect any part of the gastrointestinal tract, from the mouth to the anus, while ulcerative colitis predominantly affects the colon. Both illnesses are characterized by inflammation, impact digestive system function (Seyedian et al., 2019). Substantially influence patient quality of life. While the exact causes remain unknown, disease progression may be influenced by the composition of the gut virome. For example, both ulcerative colitis and Crohn’s disease have been associated with an increased relative sequence abundance of *Caudoviricetes* phages. In addition, phage diversity is lower on average in patients with Crohn’s disease (Pérez-Brocal et al., 2015; Fernandes et al., 2019; Khan et al., 2019; Ungaro et al., 2019).

### 4.2. Obesity and diabetes

Obesity is a chronic illness and a leading global health issue. An individual is obese if their body mass index (BMI) equals or exceeds 30 kg/m^2^ (Garvey and Mechanick, 2020). Excessive fat storage caused by metabolic, genetic, and external factors such as drug intake triggers obesity. The gut microbiome contributes approximately 8% of resting energy expenditure in mammals, and alterations in the gut microbiome cause an increase in weight gain over several months (Riedl et al., 2021). Viral infection may precede adult obesity (Hasan et al., 2021; Tarantino et al., 2021), however, it is not clear whether a specific viral assault causes obesity or a general dysbiosis results in reduction of energy expenditure and thus weight gain (Meldrum et al., 2017; Bikel et al., 2021; Riedl et al., 2021). There are also considerable changes in the virome, especially the phageome, within the gut of individuals with obesity and both type I diabetes (Zhao et al., 2017) and type II diabetes (Ma et al., 2018), but causation is not yet clear.

C57BL/6 mice are a common inbred laboratory mouse strain widely used as a model organism to investigate diabetes, obesity, and other metabolic disorders. These mice display characteristics of type 2 diabetes, such as abnormal glucose metabolism and microbial composition. Fecal virome transplantation (FVT) has been shown to alleviate those symptoms, and alter the gut microbiome reducing the onset of obesity in mice fed with high-fat diets (Rasmussen et al., 2020; Schulfer et al., 2020; Borin et al., 2023). It is possible that this reflects a shift in the viral population from virulent to temporal phages (Kim and Bae, 2016) or an reparation of lost phages that control the microbial communities (Cervantes-Echeverría et al., 2023).

### 4.3. Pregnant women with type 1 diabetes

Pregnancy is a complex physiological state that requires a stable homeostatic environment, including a vulnerable immune system. This finely-tuned setup can be disrupted by infection (Tagliapietra et al., 2020), with consequences for both the mother and the growing child. Yue et al. (2018) undertook a meta-data analysis of 25,846 participants and suggests an association between the instance of type 1 diabetes in children and maternal infection while *in utero*, finding that maternal infection is associated with a 32% increase in diagnoses of type 1 diabetes of islet autoimmunity in the child (Yue et al., 2018). Furthermore, women who have type 1 diabetes have been shown to possess a distinct gut viral profile during pregnancy with an increase in relative abundances of tobamoviruses and picobirnaviruses in the guts of women with type 1 diabetes (Wook Kim et al., 2019). This could be used as a biomarker of immunosuppression that is associated with pregnancy, in women with type 1 diabetes. However, far more extensive research is required as *Tobamoviruses* are plant viruses passing through the gastrointestinal tract, and *Picobirnavirus* is a double-stranded RNA virus that has been speculated to infect prokaryotes. This is speculative and more research is required to identify the host of the *Picobirnavirus* (Krishnamurthy and Wang, 2018) and whether these viruses could be used as biomarkers of immune suppression.

### 4.4. COVID-19/SARS-COV-2

Severe acute respiratory syndrome coronavirus 2 (SARS-CoV-2) is an enveloped RNA virus that causes coronavirus infection disease 2019 (COVID-19) a highly contagious disease of intense global interest, that affects multiple organs, including the lungs and gastrointestinal tract (Cao et al., 2021; Sanyaolu et al., 2021). Patients suffering from COVID-19 have reported symptoms such as lack of taste and smell, body aches and diarrhea, interestingly some reports have indicated altered viromes (D’Amico et al., 2020; Wang et al., 2020). A review by D’Amico et al. (2020) described how the angiotensin-converting enzyme 2, better known as Ace2, is present in the lungs and small intestines, allowing for SARS-CoV-2 to enter the eukaryotic cells and cause damage. The Ace2 enzyme is a key enzyme in the renin-angiotensin system (RAS) which is involved in acute lung failure, cardiovascular function and SARS infection (Hashimoto et al., 2012). Reports identified decreased expression of Ace2 was associated with small bowel inflammation and more severe disease of COVID-19, which could be linked to the instances of diarrhea reported in patients (Potdar et al., 2020).

Patients also showed an enrichment of environment-derived eukaryotic DNA viruses, and a decrease in diversity of the DNA virome as a whole, with a significantly reduced Shannon diversity in the gut virome, inflammation, and virulence-associated gene encoding capacities when compared to healthy controls (Lamers et al., 2020; Cao et al., 2021; Patankar et al., 2021). The decrease in virome DNA might be due to the negative impacts that COVID-19 has on the microbiome which can be associated with the Ace2 receptor described above or the treatment provided to patients suffering from COVID-19 such as antivirals and antibiotic therapy (Aktas and Aslim, 2020). This change in virome occurs not only in moderate and severe cases of COVID-19, but in non-severe and asymptomatic cases as well, and these changes can persist for 30 days after disease resolution (Zuo et al., 2021). The short-term impacts of COVID-19 on the gut virome is currently well-studied. It remains to be seen what impact COVID-19 has on the gut virome in the long term, and in patients suffering from long covid.

### 4.5. Impact of antibiotic treatment

When phage-infected microbes are lysed by antimicrobials, the phages often undergo significant changes in abundance and replication as the phages switch from a lysogenic to a lytic lifestyle, and kill their bacterial hosts (Sutcliffe et al., 2021). We know that, during a course of broad-spectrum antibiotics, diversity in bacteria decreases, while viral diversity initially increases as new viruses are released, before decreasing in response to the loss of their hosts. Interestingly, the oral ingestion of antibiotics can induce prophages to replicate and kill their host; this process has been reported in the bacteria that reside in humans (Kang et al., 2021) and swine (Allen et al., 2011; Johnson et al., 2017).

A report by Abeles et al. (2015), showed that the oral and fecal virome respond to prolonged antibiotic treatment differently, specifically the oral virome is more diverse than the fecal virome post antibiotics. Furthermore, the fecal virome contained an elevated abundance of antibiotic resistance genes that were homologous when blasted to the Comprehensive Antibiotic Resistance Database (CARD) compared to the controls (Abeles et al., 2015). These homologous genes hit a number of known resistance mechanisms including beta-lactams, vancomycin, tetracyclines and multidrug transporters. Another paper looking at the increase of antibiotic resistance genes of phages during antibiotic therapy suggests that there is an increase of resistance genes starting on the 3rd day of treatment (Górska et al., 2018). These studies and others suggest that the resistance markers in phage could be contributing to the antibiotic resistance seen in the microbial community within a patient (Modi et al., 2013). However, it is worth noting that phages rarely code antibiotic-resistance genes (Enault et al., 2017), rather they are responding to DNA damage and other cytotoxic effects of the antibiotics.

### 4.6. Fecal microbiota transplants

Fecal microbiota transplants (FMT) is the process in which the fecal microbiota of one person is transferred to another individual, to improve the levels of ‘healthy’ microbes in the recipient. FMT is often used as a method of treating gut dysbiosis and has proven to be especially effective for recurrent *C. difficile* infections (Ramay et al., 2016; Conover et al., 2023). Treatment results in long-term changes to the recipient’s gut microbiome and virome (Broecker et al., 2016a; Draper et al., 2018), and the phages transferred during an FMT can play a critical role in shaping treatment outcome (Zuo et al., 2018).

We now know that phages modulate gut microbiota and its function, this treatment could be further specialized to transplanting the virome, called fecal virome transplant (FVT). Trials involving (FVT), filter the donated fecal material to remove the bacteria and larger components leaving only the viruses, metabolites, proteins and other small particles for the transplant. Studies have shown that FVT has similar results as FMT for treating *C. difficile* infection, with phages positively associated with treatment outcomes (Ott et al., 2017; Zuo et al., 2018; Lin and Lin, 2021). FVT’s have also effectively decreased the symptoms of type 2 Diabetes in mice, decreasing weight gain, and altering the microbial community (Draper et al., 2020; Rasmussen et al., 2020), and have helped to restore the microbiome to its previous state following antibiotic disruption of the gut microbiome in mice (Draper et al., 2020).

## 5. Possible virome effects on psychological health

### 5.1. The gut as a second brain

The gut-brain axis is described as a bi-directional system connecting the brain and the gastrointestinal tract (Jenkins et al., 2016; Bonaz et al., 2018). The main communication pathways are between the central nervous system and the peripheral nervous system involving the vagus nerve (stomach and rectum), the dorsal root ganglia (small and large intestines) and the gut nervous system (ENS) to the sympathetic ganglia and parasympathetic nervous system (Jenkins et al., 2016; Strandwitz et al., 2019; Haas-Neill and Forsythe, 2020). Bacteria probably do not directly interact with the gut-brain axis but produce several molecules that do interact, known as neurochemical-based interkingdom signaling. The molecules produced include the same neurohormones found in the host (Lyte, 2014), short-chain fatty acids like gamma-amino butyrate which directly influence the release of the neurotransmitter serotonin from enterochromaffin cells (Kaur et al., 2019) and the release of acetylcholine and glucagon-like peptide −1 and peptide YY from enteroendocrine cells (Bonaz et al., 2018; Haas-Neill and Forsythe, 2020). A few bacterial species such as *Streptococcus*, *Enterococcus,* and *Escherichia* can synthesize serotonin, dopamine and norepinephrine, however the microbe-derived host responses are not well characterized (Cryan et al., 2019; Kaur et al., 2019). Neurons of the ENS system that innervate the intestinal mucosa could act as the initial neural detectors of signals from the microbiota, which then modulate vagal activity through synaptic transmission (Breit et al., 2018). In essence, the gut-brain axis uses this interkingdom signaling between the gut bacteria and potentially the entire microbiome, nerve cell membrane vesicles and the host endocrine, immune, and neural signaling pathways to link emotion and cognitive behaviours of the host with microbial gastrointestinal residents, including the virome (Jenkins et al., 2016; Haas-Neill and Forsythe, 2020). Eukaryotic viruses such as norovirus, rotavirus, and coronavirus can cause gastroenteritis (inflammation of the lining of the intestines), which can lead to changes in the gut microbiota and subsequently impact the gut-brain-axis (Sajdel-Sulkowska, 2021). Respiratory viral infections can also alter the gut microbiota. There was a decrease in Firmicutes and an increase of Bacteroides following RSV infection, and a significant shift in lipid metabolism: sphingolipids, polyunsaturated fatty acids and short-chain fatty acids were all increased post viral infection (Groves et al., 2020). We know that short-chain fatty acids can play a role in interkingdom signaling, therefore, these alterations could have effects on the gut-brain axis. Other viruses, such as herpes simplex have also been shown to affect the central nervous system and cause gut microbiota dysbiosis (Li et al., 2023).

Diversity of the gut microbiota plays a significant role in the mental health of individuals via the gut-brain axis and modulates stress responses, anxiety, depressive states and memory (McGovern et al., 2019). Serotonin, a neurotransmitter that affects mood, cognition, sleep, and memory is essential in these processes (Jenkins et al., 2016; McGovern et al., 2019). There are reports of COVID-19-associated neuropsychiatric disorders including anxiety, major depressive disorder, posttraumatic stress disorder, and neurological disorders such as encephalitis. Interestingly these associations were reported independent of respiratory disease severity, potentially due to the viruses’ independent action on the gut-brain-axis (Liotta et al., 2020). Phage impact the bacteria within the gut through lysogenic and lytic relationships and there have been suggestions that gut dysbiosis of microbial species may induce atypical immune signally, an imbalance in host homeostasis and central nervous system disease progression (Ma et al., 2019). However, more work is needed to elucidate the mechanisms of action phage and other viruses have on the bacteria, intestinal lining, inflammation of the gut, and the production of serotonin. All these factors could potentially impact mental health.

### 5.2. Virome and mental health

Compared with exploration of how commensal bacterial communities impact human health, the role of the virome is relatively understudied in mental health (Yolken et al., 2021). However, there are reports that detail alterations in the virome, such as viral infections, causing inflammation in the brain affecting mental health. Research has detailed correlations between maternal exposure to bacterial and or viral infections during pregnancy such as influenza, rubella or herpes and brain function developmental changes in the offspring (Comer et al., 2020). Exposure to a bacterial or viral infection elevates the levels of pro-inflammatory cytokines and this elevation has been associated with increased risk of developing schizophrenia (Comer et al., 2020; Kępińska et al., 2020).

We know that more than half of the patients with IBD suffer from mood disorders, and antidepressants have been listed as one of the most common pharmaceutical interventions for patients with inflammatory bowel syndrome (Rogers et al., 2016; Bernstein, 2018). This latter intervention leads to an altered composition of the gut microbiota, suggesting that the microbiome and perhaps the virome itself plays a role in mood disorders (Bonaz et al., 2018; Lukić et al., 2019). There are three bacterial genera in the gut –– *Ruminococcus*, *Adlercreutzia*, and an undefined genus in the order RF32 (class Alphaproteobacteria) –– that are affected by antidepressants, this along with our understanding that patients suffering from IBD already have an altered enteric virome (Section “Inflammatory bowel disease”), we presume, phages that infect these bacteria will also be affected by medication (Lukić et al., 2019). This is a complex situation to unravel because patient mental health depends not only on an altered microbiome and the specific virome but also symptoms of disease itself. Separating the contribution of each component on mental health is not straightforward and will require careful study design and a holistic approach for translation into health care.

## 6. Bioinformatics

### 6.1. Bioinformatic challenges of viral dark matter

There are many bioinformatic challenges with viral metagenomics, and these are mostly related to viral dark matter. While viruses are the most abundant organism on the planet, they are severely underrepresented in reference databases. The knowledge that there still exists an amazing diversity of viral species which we have yet to study has led to the description of this great unknown as viral dark matter. As a result, viral dark matter represents a tremendous opportunity for discovery, it has been estimated that viral dark matter comprises 40–90% of the total expected gut viruses in a sample (Krishnamurthy and Wang, 2017), although this fraction may vary due to biases introduced by experimental and computational methods and study specific differences (Santiago-Rodriguez and Hollister, 2020). For example, a study across 6,000 diverse environments was able to annotate only 5.1% of phage proteins to proteins with known function (Paez-Espino et al., 2016). Meanwhile, in the human gut, matches have been found for 50% of phage proteins (Elbehery et al., 2018). Although strategies have been devised to assist in reducing viral dark matter (Brum et al., 2016; Tisza et al., 2020), many challenges remain. This includes isolation, culturing, and enrichment of viruses, as well as challenges associated with the extraction of viral genetic material, biases in sequencing methods, and limitations with sequence analyses (Bowers et al., 2015; Sato et al., 2019; Sutton et al., 2019). Nevertheless, recent years have seen some excellent progress with both wet-lab protocols and bioinformatics advancements which we discuss.

### 6.2. Lab-grown bioinformatic challenges

The quality of bioinformatic analysis is intricately tied to the experimental approaches. The traditional and ideal ‘targeted approaches’ that isolate, culture, and sequence a pure clonal population of a virus will result in the best reference genome assemblies. However, not all microscopic organisms can be cultured, and those that can are typically only a fraction of the total diversity *in vivo*. New research has implemented broad-rang culturing with media such as YCFA to alleviate these issues (Browne et al., 2016), these methods have increased the proportion of culturable bacteria but are not able to capture everything. An untargeted –viral metagenomics –approach is therefore needed to capture the complete virome and to uncover the sheer breadth of diversity that exists among viruses (Górska et al., 2018). This approach requires extraction, and sequencing of the viral genetic material from fecal samples, followed by computational analysis using bioinformatics tools to characterize these viral species.

Sample protocols can cover specific niches, for instance, extraction and amplification of low-volume samples, whereas other protocols may have a broader scope (Shkoporov et al., 2018b; Deng et al., 2019). Improper sample handling and storage and sample processing for viral purification and enrichment can all result in the loss or degradation of viral genetic material, resulting in skewed or absent viral species (Cantalupo and Pipas, 2019). Procedures may allow for rigorous analysis across datasets, but unfortunately, various methods are currently published that use different extractions kits and slightly different approaches, each resulting in a different profile of extracted viruses due to the binding capacity of column kits as well as other influences such as water pH; with no one method being objectively superior (Hall et al., 2014; Conceição-Neto et al., 2015; Kleiner et al., 2015). A typical process is undertaken for virome DNA extractions. It begins with pre-processing of the sample, which involves homogenization in a buffer that supports maintaining the integrity of the virome through the extraction process. SM buffer is recommended throughout the literature, made up of NaCl, MgSO4, Tris–HCL, and 2% gelatin.

When working with fecal samples, the samples are homogenized via vortexing, which helps release the virome from the fecal material (Thurber et al., 2009; Maghini et al., 2021). Centrifugation is then undertaken, followed by pelting the larger fecal material. The supernatant is collected and filtered through either a 0.45 or a 0.22 μm syringe removing the bacteria from the sample. The filtrate is the virome lysate and can now be lysed and DNA extracted. The PEG and cesium chloride methods are common and have been used widely (Deng et al., 2019; Adiliaghdam et al., 2022; Lee et al., 2022); however, these are time-consuming. Other methods include concentrating the sample prior to DNase I treatment to remove the host genetic material (Thurber et al., 2009). Once the above is completed the sample is ready to be lysed and the virome DNA extracted. This process can be undertaken by phenol/chlorophore standard methods and followed by ethanol precipertation. However, many kits can perform these tasks quickly. For example, Norgen, Qiagen, and Macherey Nagel produce kits to extract viral DNA. Ultimately it is desirable to have concentrations higher than 2 ng/μl to send for barcoding and sequencing either through illmina or Nanopore platforms.

Many bioinformatics problems can be addressed or exacerbated by design decisions in the laboratory. For instance, whole genome amplification can aid with retrieving low-abundance viruses, but representation bias, error rates, yield, and robustness can affect downstream analyses (Deleye et al., 2017). Different sequencing technologies can impact the observed abundances. Low-abundance species can be biologically or clinically significant, but inadequate sequencing depth can result in such low-abundance species not being captured. Finally, improper sequence processing and analysis can result in important viruses being filtered or remaining unannotated (Cantalupo and Pipas, 2019; Martínez-Puchol et al., 2020). Prior to laboratory experiments, appropriate planning of experimental design is highly recommended to ensure adequate sample size and that the sequencing approach is suitable for the bioinformatic objectives.

### 6.3. Our dependence on databases

Following extraction and sequencing of gut viruses, computational analyses are applied to filter viral sequences from non-viral genetic material and to perform searches against established datasets of known viral sequences (Arndt et al., 2016; Ren et al., 2017; Zheng et al., 2019). Direct annotation of sequencing reads can be a computationally efficient way of exploring and quantifying the viral sequences in a metagenome. However, the speed and performance of these analyses are entirely dependent on the quality and size of the reference databases of known viral sequences. There are approaches that do not entirely depend on databases for viral annotations such as Virsorter2 (Guo et al., 2021), however, these still depend on the databases, for instance in training the machine learning classifiers. Specialized databases are the logical solution to balancing efficiency with performance. Two of the largest specialized gut virome datasets are the “Human Gut Virome Database, GVD,” which encompasses 13,203 viral populations which approximate the species-taxa level (Gregory et al., 2020), and the “Gut Phage Database, GPD,” which encompasses 142,809 phages (Unterer et al., 2021). Other recent efforts have produced 189,680 viral metagenome-assembled genomes (MAGs; Nayfach et al., 2021b), and the latest version of IMG/VR contains over 15 million MAGs (Camargo et al., 2023). Unfortunately, the reliability of these viral MAGs is uncertain and as such they are often not included in the popular reference databases, such as GenBank or RefSeq, which are typically used for annotating metagenomes. Furthermore, GenBank contains only 4,509 phage genomes (National Center for Biotechnology Information (NCBI), 1988), and these are prone to mislabelling. Conversely, the use of the NCBI non-redundant database allows comparisons with all known sequences, albeit at the cost of significantly increased computational complexity. Novel methods such as the viral metagenomics platform Hecatomb (Roach et al., 2021, 2022) and Phables (Mallawaarachchi et al., 2023) are emerging as a solution to this issue. Hecatomb^1^ produces viral annotations using a specialized database and an iterative search strategy. It is designed to be computationally efficient, while maintaining a highly sensitive search strategy that can identify viruses that may only be distantly related to sequences in the reference databases. The iterative strategy further removes most false positive hits that are likely to arise which greatly simplifies downstream analysis. Hecatomb also includes a cross-assembly that can be combined with Phables^2^ to generate complete, high-quality phage genomes from metagenome samples.

### 6.4. The problem with prophages

The prominence of prophages in gut viromes brings about its own set of challenges. Prophages can bring about drastic changes in bacteria by supplying genes that confer useful phenotypes (Schroven et al., 2021). For example, phage genes may aid the bacteria in cellular replication or prevent further infection from other phages through superinfection exclusion mechanisms (Dulbecco, 1952; Lu and Henning, 1994; Owen et al., 2020). Some phage genomes also confer additional virulence factors or antimicrobial resistance genes upon integration into the bacterial genome (Marti et al., 2014; Edwards et al., 2019). Indeed, most bacterial exotoxins involved in disease pathogenesis are encoded on phage genomes (Wagner and Waldor, 2002; Dydecka et al., 2017). These integrated phages present an interesting challenge in understanding the phageome of the gut. A wide range of bioinformatics tools have been developed to find phages that may be hiding within bacterial genomes with varying degrees of success (Roach et al., 2021).

Predicting prophage regions in bacterial genomes remains a challenging task. While some tools such as PhiSpy, Phigaro, VIBRANT, and CheckV appear to work reasonably well, many computational analyses are simply unable to reliably distinguish prophage regions from the remainder of bacterial genomes (Nayfach et al., 2021a; Roach et al., 2022). This is especially problematic when considering read-based annotation of phages, where it may be impossible to determine if a read originated from a phage or a prophage region in a bacteria’s genome. Furthermore, cryptic phages–prophages that have lost the ability to excise from their bacterial genome–can further confound phage annotations. For instance, should they be annotated as bacterial because they can no longer replicate as a phage, or as prophages due to their origins? As temperate phages can constitute a majority of the viruses within the human gut (Sausset et al., 2020), this presents a significant challenge for gut viral metagenomics.

Cultivating high-quality viral, phage, and prophage sequences and submitting them to the standard reference databases will be incredibly important in the coming years. Current efforts are underway to mine prophage sequences from existing bacterial genomes using best-in-class algorithms (Akhter et al., 2012; Roach et al., 2021, 2022). These will help to build the knowledge base that is required for correctly classifying prophages and temperate phages in metagenome samples.

## 7. Conclusion

The establishment of the gut virome begins at birth. Method of delivery has an observable impact on the constituents of an individual’s gut virome. The gut virome is further modified in early life by the mode of feeding, with oligosaccharides in breast milk playing an essential role in the development of the gut flora. The gut virome becomes stable over time, is highly specific to each individual, and relates to various aspects of human health. The virome is impacted by a broad swathe of factors, including but not limited to age, diet, disease states, and antibiotic treatments.

The gut virome is composed primarily of phages. These viruses play an important role in controlling the mortality, and composition of the microbiome, which, when considered together, play a critical role in human health. More studies are required to examine gut dysbiosis’s effects on the core virome and the phage population. Restoring core, stable gut microbiome species through FMT and/or FVT can positively impact health and quality of life, improving the functionality of the gut after illness and as an effective agent for treating disease.

The existence of viral dark matter is a perplexing problem that still plagues biologists after over 100 years since phage discovery. One of the challenges is the inability to culture and isolate many of these species *in vitro*, which has been addressed bioinformatically using metagenomics and advanced sequencing methods. The bioinformatic programs that mine viral dark matter are adopting new strategies and increasing functionality, speed, and effectiveness. Several new phages, including crAss-like phages, gubaphage, and Lak phages have been found from directly mining metagenomic data. The gut virome is a complex, highly faceted, and essential feature that contributes to maintaining human health.

## Author contributions

EP, RE, and SKG: conceptualization. EP, MR, AS, RE, and SKG: writing–original draft. EP, MR, AS, BP, LI, VM, SRG, CH, RE, and SKG: writing–review and editing. All authors contributed to the article and approved the submitted version.

## Funding

This work was supported by an award from NIH NIDDK RC2DK116713 and an award from the Australian Research Council DP220102915.

## Conflict of interest

The authors declare that the research was conducted in the absence of any commercial or financial relationships that could be construed as a potential conflict of interest.

## Publisher’s note

All claims expressed in this article are solely those of the authors and do not necessarily represent those of their affiliated organizations, or those of the publisher, the editors and the reviewers. Any product that may be evaluated in this article, or claim that may be made by its manufacturer, is not guaranteed or endorsed by the publisher.

## Footnotes

^1^github.com/shandley/hecatomb

^2^github.com/Vini2/phables
